# Composite Classical Hodgkin Lymphoma and Mantle Cell Lymphoma: A Case Report

**DOI:** 10.7759/cureus.45727

**Published:** 2023-09-21

**Authors:** Rajdeep Kaur, Bingjun Zhang, Kuixing Zhang, Mohamed Eldaly, Jincy Clement

**Affiliations:** 1 Hematology and Medical Oncology, Emanuel Cancer Center, Turlock, USA; 2 Biochemistry, University of California (UC) Davis, Davis, USA; 3 Pathology and Laboratory Medicine, Emanuel Medical Center, Turlock, USA

**Keywords:** rchop therapy, reed-sternberg cells, mantle cell lymphoma, classical hodgkin lymphoma, hodgkin's lymphoma non-hodgkin's lymphoma

## Abstract

Composite lymphoma implies the presence of two or more morphological and immunophenotypical subtypes of lymphoma in a single tissue or organ. Composite lymphoma with concurrent mantle cell lymphoma (MCL) and classical Hodgkin lymphoma is extremely rare. In this case report, we present the case of a 70-year-old male who was diagnosed with a composite of MCL and classical Hodgkin lymphoma (cHL) and achieved near-complete resolution with chemoimmunotherapy. To the best of our knowledge, this is the first case of this kind demonstrating the effectiveness of a combination chemoimmunotherapy regimen leading to complete remission in composite lymphoma involving MCL and cHL. We report the history, imaging findings, and pathology and illustrate the challenges in therapeutic decision-making in managing composite lymphoma patients involving MCL and cHL. We also review the literature on this rare entity and discuss its clinical implications.

## Introduction

Composite lymphoma is a rare condition that occurs when two or more distinct types of lymphoid neoplasms coexist in the same anatomic location. The incidence of composite lymphoma is low, ranging from approximately 1.0%-4.7% of all lymphomas. Various combinations of composite lymphoma have been reported, but the most common scenario is the concurrent occurrence of two non-Hodgkin lymphoma (NHL) subtypes. The synchronous presentation of NHL and Hodgkin lymphoma (HL) is less frequent, and the composite of mantle cell lymphoma (MCL) with classical HL (cHL) is extremely rare. To the best of our knowledge, only 11 cases have been reported in the literature since 2003 [1-9].

The pathogenesis and clinical behavior of composite lymphoma are poorly understood, and there are no established guidelines for the diagnosis and treatment of this rare entity. The prognosis of composite lymphoma may depend on the aggressiveness and responsiveness of each component, as well as the patient’s characteristics and comorbidities. Therefore, each case of composite lymphoma represents a unique challenge and an opportunity to learn more about this complex disease.

## Case presentation

**Figure 1:**
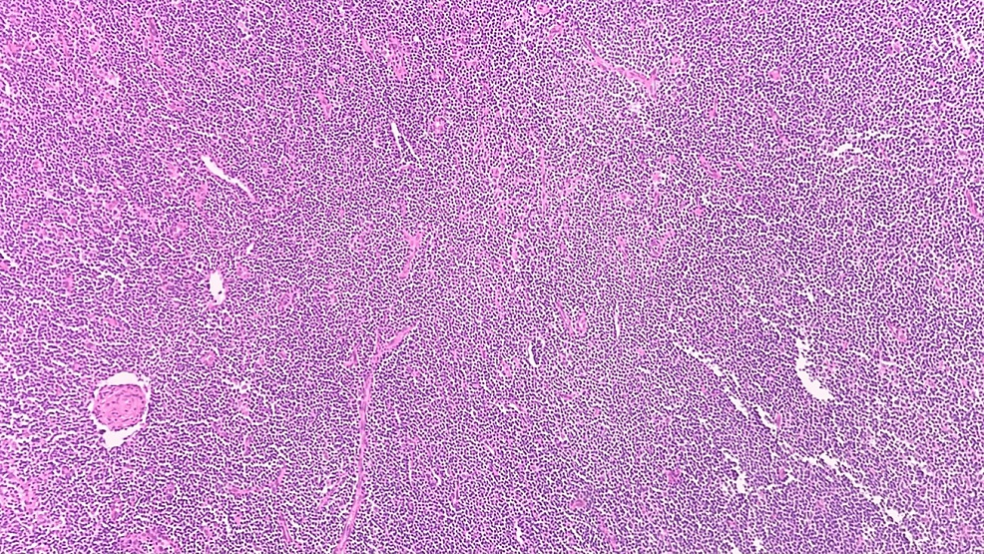
Right neck mass​ H&E stain, 100x, showing proliferation of small lymphocytes​ H&E: Hematoxylin and eosin.

**Figure 2:**
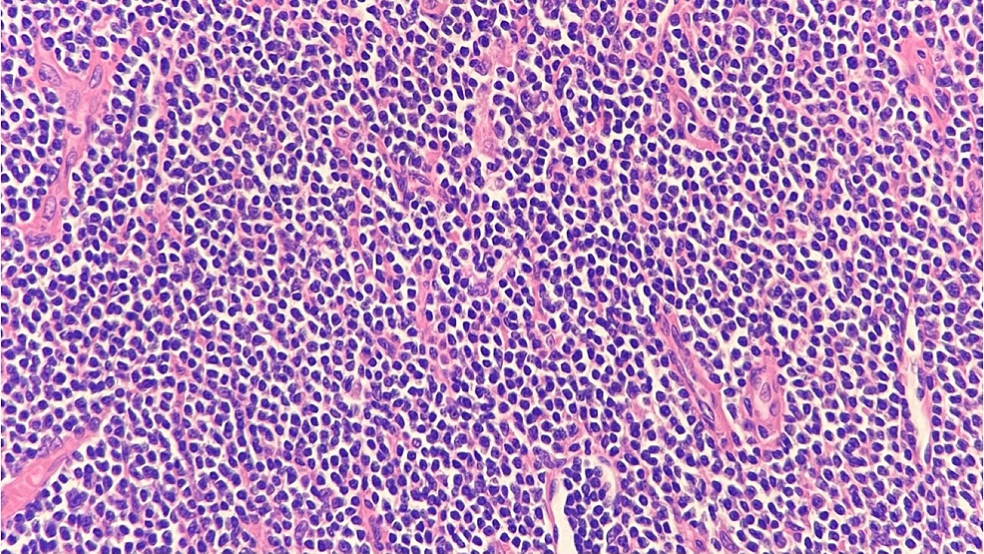
Right neck mass H&E stain, 400x, showing small, homogenous lymphocytic proliferation H&E: Hematoxylin and eosin.

**Figure 3:**
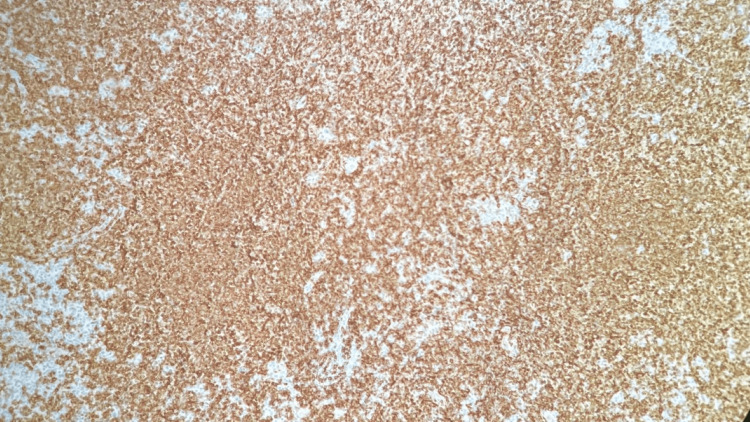
Right neck mass CD20 stain, 100x, highlights the B lymphocytes

**Figure 4:**
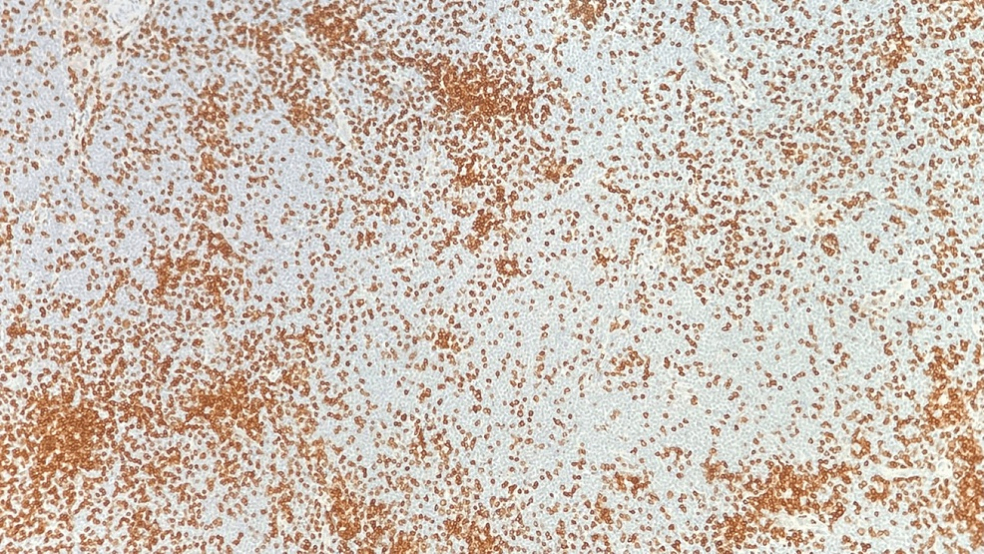
Right neck mass CD3 stain, 100x, highlights the background T lymphocytes

**Figure 5:**
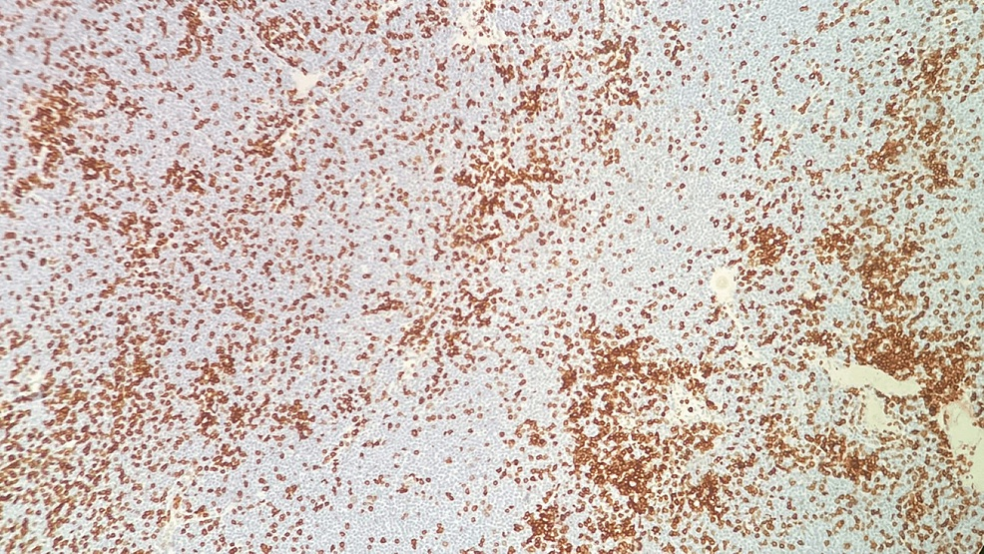
Right neck mass CD5 stain, 100x, negative for B cells and positive for background T cells

**Figure 6:**
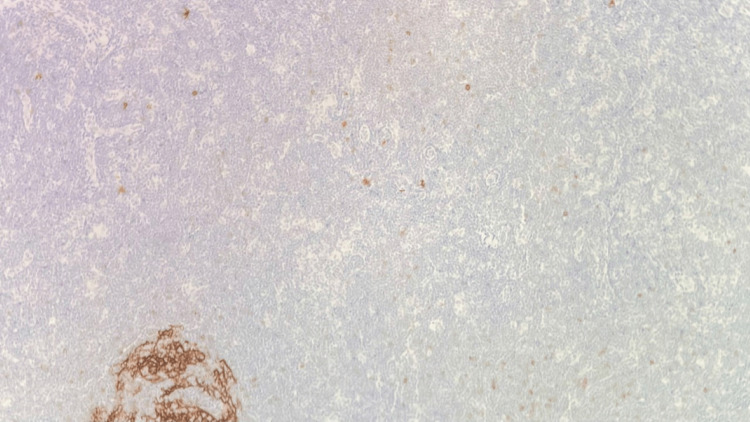
Right neck mass LEF1 stain, 100x, negative for B cells and positive for background T cells LEF1: Lymphocyte enhancer–binding factor 1.

**Figure 7:**
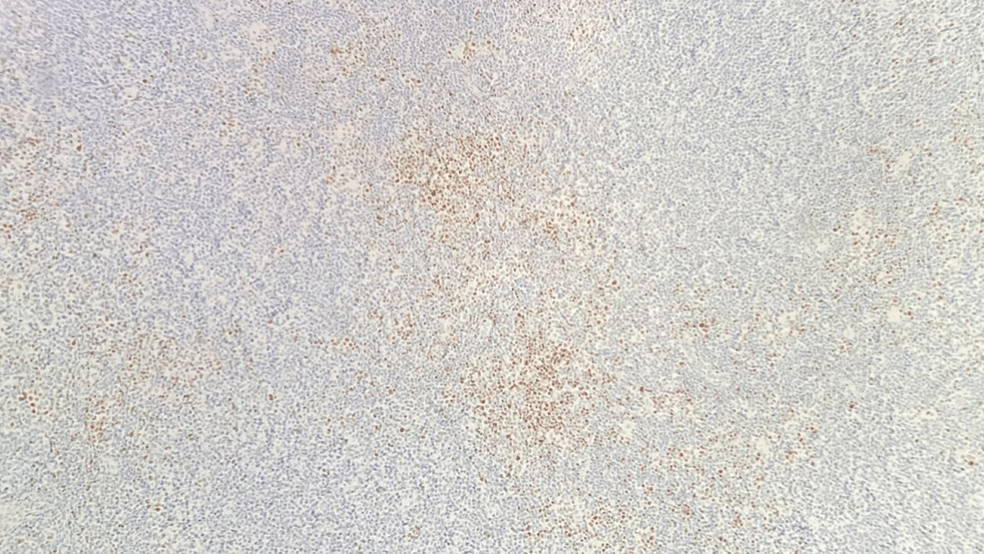
Right neck mass SOX11 stain, 100x, negative

A 70-year-old male presented with a gradual-onset right neck mass that had been evolving over a four-month period. He denied experiencing nocturnal fever, night sweats, chills, weight loss, or other B symptoms. His laboratory parameters were unremarkable except for hyperglycemia consistent with his medical history. An ultrasound revealed a right neck mass measuring 26 mm x 22 mm x 7 mm. Subsequently, an excisional biopsy of the adjacent lymph node was performed, and pathological examination indicated an atypical lymphoid cell infiltrate positive for CD20, CD45, BCL1, and BCL2 while testing negative for CD3, CD43, CD5, CD10, BCL6, LEF1, and SOX11. The Ki-67 proliferation index was 5%-10%. Fluorescence in situ hybridization (FISH) analysis confirmed the presence of the t(11:14) translocation. Flow cytometry revealed a lambda-restricted B cell population expressing CD19 and CD20 while testing negative for CD5, CD10, CD11c, and CD38. These findings collectively supported a diagnosis of CD5-negative MCL (Figures 1-7).

A positron emission tomography scan-computed tomography (PET-CT) was performed for staging and evaluation, which revealed extensive cervical lymphadenopathy more pronounced on the right side. The involvement included the right level II A and B, level III, and level IV lymph node regions, along with the posterior triangle. Notable findings included a lymph node up to 25 mm in length with a standard uptake value of 6.4. A moderately hypermetabolic lingual tonsil uptake (21 mm, Maximum Standard Unit Value [SUVmax]: 5.3) was observed to the right of the midline. In the axillary region, lymph nodes on the left (up to 3.7 cm) and right (up to 3.2 cm) exhibited standard uptake values of 3.6 and 2.1, respectively. Splenomegaly was noted, with a spleen size of 16.6 cm, yet spleen uptake remained within the normal range.

Given the patient's asymptomatic status with no cytopenia and the context of the ongoing pandemic, a decision was made to initiate observation with continuous monitoring.

Over the subsequent months, the patient's lymphadenopathy exhibited minimal change on serial PET-CT scans, and he remained asymptomatic.

However, during continuous monitoring of nearly 1.5 years, a PET-CT scan revealed a significant increase in the size and standard uptake of multiple confluent lymph node groups, indicative of disease progression. Notably, the right jugular chain exhibited the largest node group, displacing the overlying sternocleidomastoid muscles. The dimensions measured approximately 10.3 cm x 4.2 cm x 4.5 cm, with a standard uptake of 9.74 SUVmax. Additionally, the left axillary region demonstrated bulky hypermetabolic adenopathy involving levels I, II, and III axillary nodes. Max SUV reached 12.5, and dimensions approximated 12.7 cm x 6.5 cm x 6.5 cm. Bulky right cervical adenopathy was also evident. Extensive periaortic and pelvic adenopathy, as well as newly developed paratracheal and subcarinal adenopathy, signaled disease progression both above and below the diaphragm. Stable splenomegaly was maintained, but new lytic hypermetabolic lesions were identified in the T2 and T10 vertebral bodies.

**Figure 8:**
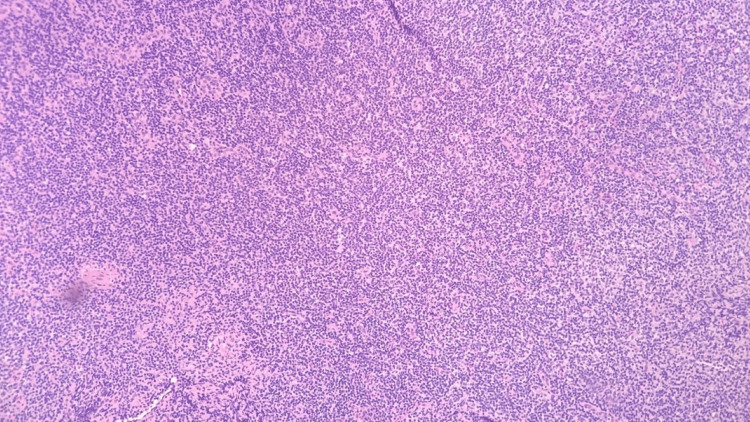
Right neck lymph node biopsy H&E stain, 100x, showing large Reed-Sternberg-like cells in the background of small lymphocyte proliferation H&E: Hematoxylin and eosin.

**Figure 9:**
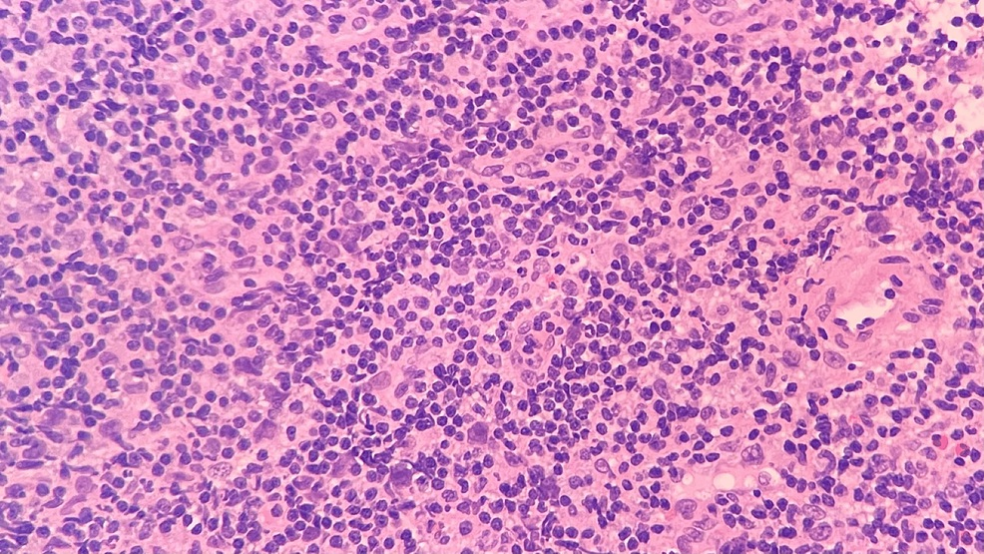
Right neck lymph node biopsy H&E stain, 400x, showing large Reed-Sternberg-like cells in the background of small lymphocyte proliferation H&E: Hematoxylin and eosin.

**Figure 10:**
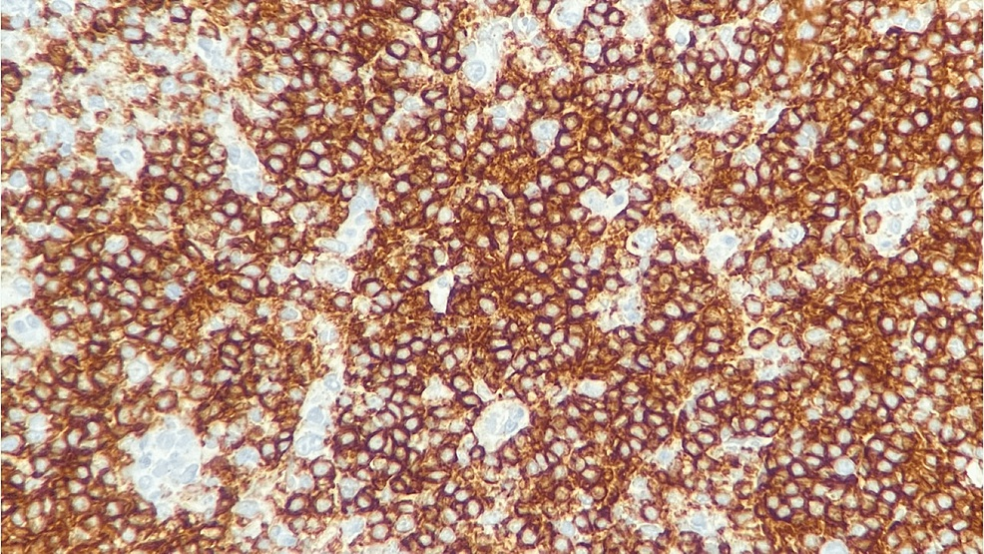
Right neck lymph node biopsy CD20 stain, 400x, positive for small lymphocytes and negative for large Reed-Sternberg-like cells

**Figure 11:**
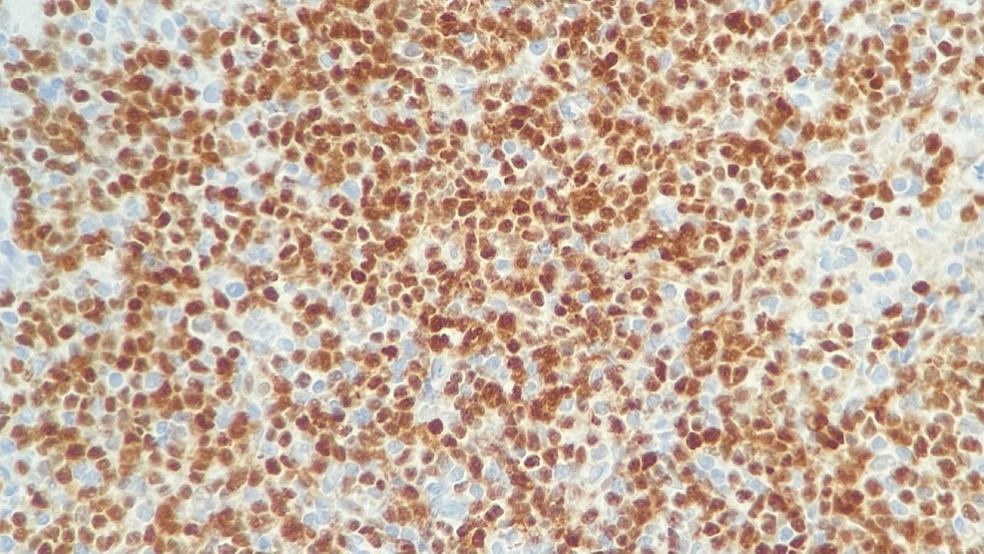
Right neck lymph node biopsy BCL1 (Cyclin D1) stain positive for small lymphocytes and negative for large Reed-Sternberg-like cells

The excisional biopsy of the right neck lymph node demonstrated total effacement of the lymph node by a predominance of small lymphocytes. Interspersed among these lymphocytes were large, atypical cells exhibiting mono- and binucleation, vesicular chromatin, and prominent macro nucleoli, characteristic of Hodgkin/Reed-Sternberg cells. Immunohistochemical stains revealed the large, atypical cells to be positive for CD30 (bright, uniform), CD15 (bright uniform membranous and Golgi), and dim Pax5 while testing negative for EBER, CD20, CD3, CD5, CD10, Alk1, and SOX11. The atypical small lymphocytes were positive for CD20, BCL1, and BCL2 but negative for CD5, CD10, and EBER. CD23 highlighted residual follicular dendritic cell meshwork. The pathology findings were consistent with CD5-negative MCL with classical Hodgkin/Reed-Sternberg-like cells, supporting the diagnosis of composite lymphoma involving MCL and cHL (Figures 8-11).

The patient began experiencing significant B symptoms, including low-grade fever and increased swelling in the right neck mass. Given the disease's aggressiveness and worsening symptoms, a decision was made to initiate chemoimmunotherapy with an R-CHOP regimen containing Rituximab, cyclophosphamide, doxorubicin hydrochloride (hydroxydaunomycin), vincristine sulfate (Oncovin), and prednisone.

**Figure 12:**
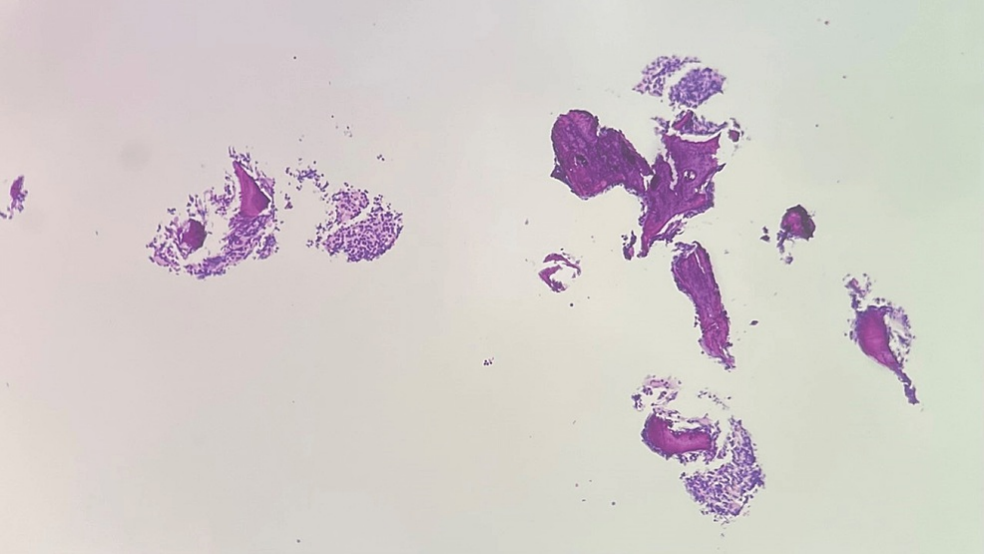
T2 vertebral bone biopsy H&E stain, 100x, showing the plasma cell proliferation H&E: Hematoxylin and eosin.

**Figure 13:**
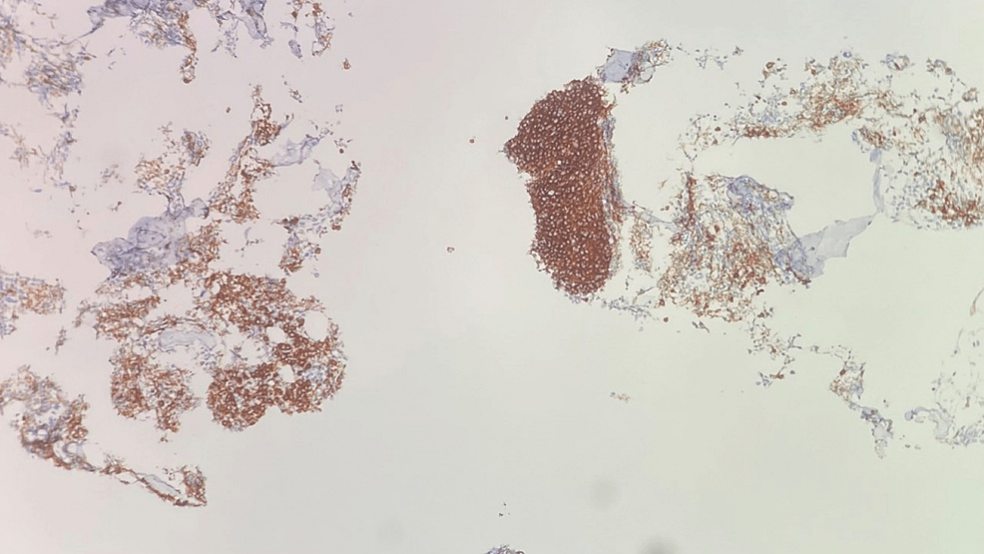
T2 vertebral bone biopsy CD138, 100x, highlighting the plasma cell proliferation

**Figure 14:**
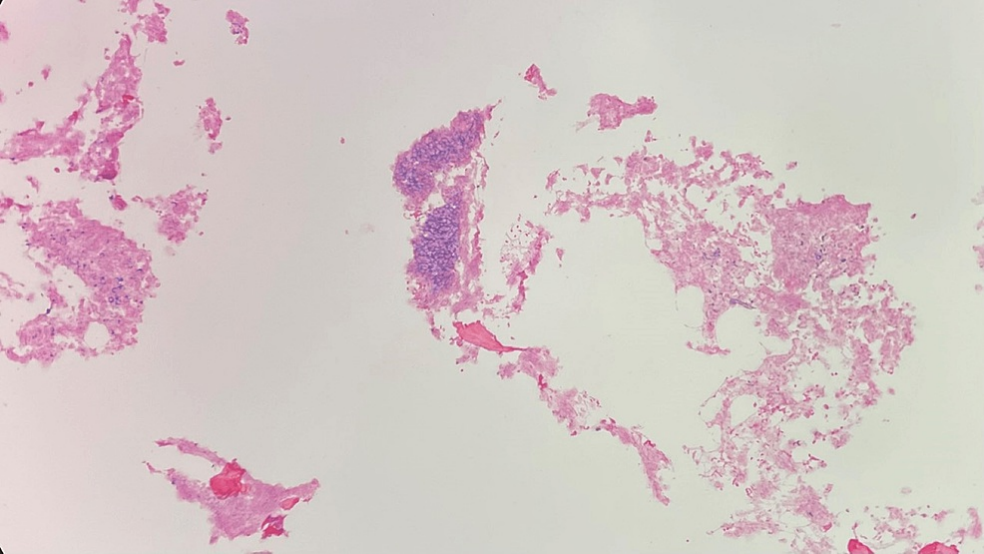
T2 vertebral bone biopsy Lambda in situ hybridization (ISH) stain, 100x, highlighting the predominant plasma cells with lambda light chain

**Figure 15:**
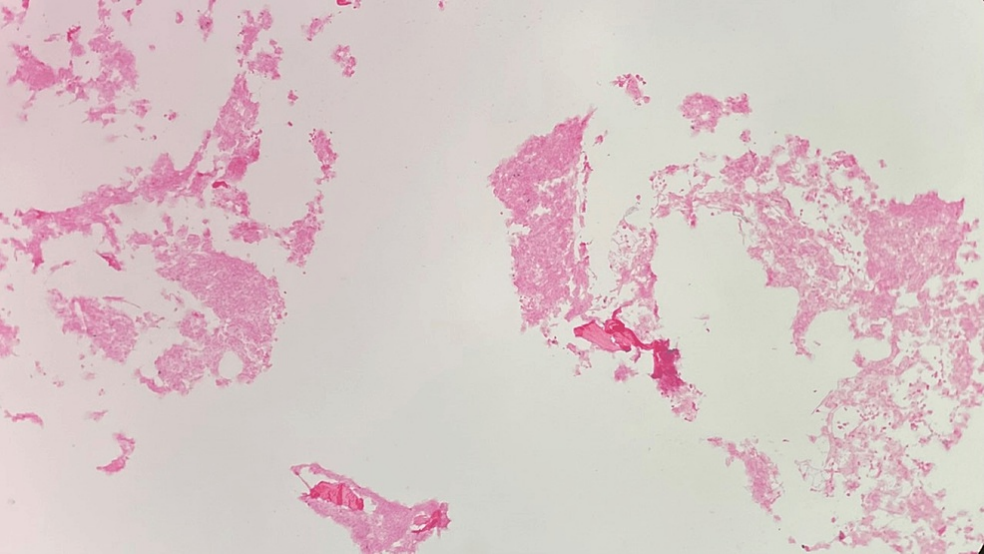
T2 vertebral bone biopsy Kappa in situ hybridization (ISH) stain, 100x, highlighting the rare background plasma cells with Kappa light chain

**Figure 16:**
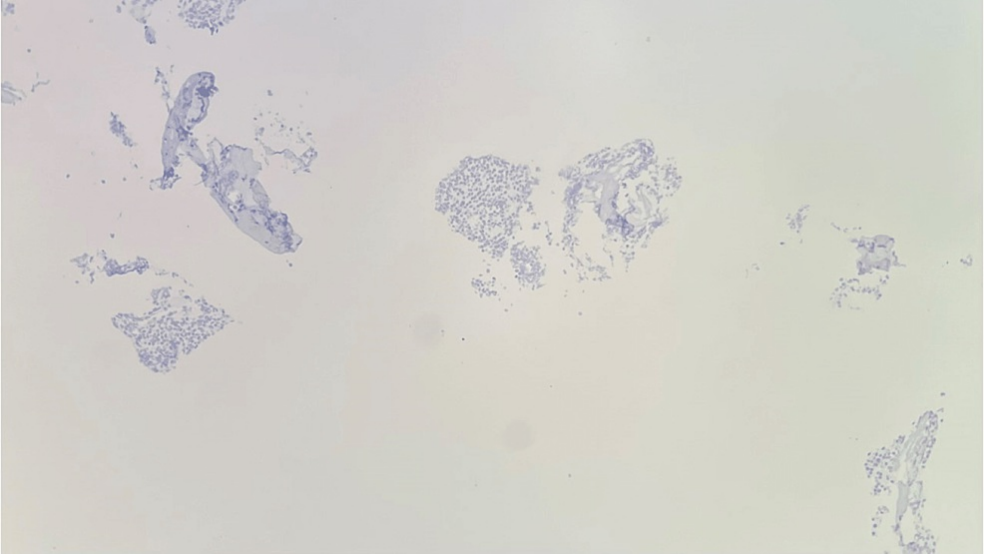
T2 vertebral bone biopsy CD30 stain, 100x, negative, no Reed-Sternberg-like cells

To further evaluate an F-fluorodeoxyglucose (FDG)-avid hypermetabolic lesion seen on PET-CT, a biopsy of the T2 vertebra was performed, revealing lambda-monotypic plasma cells with BCL1 expression. Flow cytometry indicated 5.9% lambda monoclonal B cells that were CD19 and CD20 positive but CD5 and CD10 negative. Additionally, 8.6% lambda monoclonal plasma cells were detected, showing negativity for CD56 (Figures 12-16). The findings were consistent with plasma cell myeloma and small foci of monoclonal B cells consistent with MCL involvement.

Following the completion of three cycles of R-CHOP, a PET-CT scan revealed complete regression of the extensive bulky adenopathy observed in the left axillary regions. Mildly prominent residual nodes were noted in level I, measuring up to 3 cm long, with a standard uptake value of approximately 2.5 (previously 12.5). There was also interval regression of subcarinal and right paratracheal adenopathy, with lymph node size reduced by an average of about 50%. The previously observed hypermetabolic periaortic and pelvic adenopathy had resolved, and the spleen size returned to normal.

As of the present, the patient is in good health, asymptomatic, and has successfully completed six cycles of R-CHOP chemotherapy. A subsequent PET-CT performed after the completion of treatment showed nonspecific symmetric uptake in several areas, including the palatine tonsil, nasopharynx, base of the tongue, parotid glands, and submandibular glands. No significant hypermetabolic uptake was observed. Notably, the jugular and supraclavicular lymph node regions exhibited no abnormal uptake.

## Discussion

The term "composite lymphoma" was given by Custer in 1954 [1] and was later modified by Kim et al. to the currently accepted definition [2]. Composite lymphoma implies the presence of two or more morphologic and immunophenotypic different types of lymphoma in a single tissue or organ [10]. Composite lymphomas can be combinations of two NHLs or a combination of a non-Hodgkin's lymphoma and a Hodgkin's lymphoma. The concurrent occurrence of two NHL subtypes is more common than synchronous NHL and Hodgkin’s lymphoma.

The etiology and pathogenesis of composite lymphoma are complex and variable. Several theories have been presumed including clonal selection, genomic instability, or common precursor cell [11]. The actual origin of the composite of MCL with HL is still unclear. Until now, most of the studies that reported the composite of mantle cell lymphoma with classical Hodgkin lymphoma (cHL) cases have investigated molecular changes and discovered different clonal relationships between the MCL and cHL components. Unlike other B cell lymphomas such as chronic lymphocytic leukemia/small lymphocytic lymphoma which have been described to present with Hodgkin/Reed-Sternberg-like cells (HRS) or with overt transformation to classical HL (HL Richter transformation), this phenomenon is very rarely described in MCL. Only a few cases have been published and demonstrate a heterogeneous range of findings. The diagnosis is based on an assemblage of findings from ancillary studies, which include flow cytometry, immunohistochemistry, and cytogenetic and molecular testing, which could reveal a possible concurrent neoplastic component.

The T2 vertebra biopsy presents an additional challenge in our case. The presence of a predominance of lambda-monotypic plasma cells forming a lytic lesion is suggestive of a plasma cell neoplasm. While we cannot completely exclude the fact that this represents plasmacytic differentiation of the patient's MCL, the differential is not favored due to the rarity of such cases, and clinical and serologic testing would be required to make this distinction. Additional evaluation with bone marrow biopsy is required but is deferred at this point of time given the challenge in patient compliance.

Given the rarity of the composite of MCL with HL, there are currently no agreed standards for the treatment of composite lymphomas. Therapeutic decisions should be based on the component of which malignant degree is higher. With respect to potential treatment outcomes, it is interesting to consider the outcome in patients treated with R-CHOP (rituximab, cyclophosphamide, doxorubicin, vincristine, and prednisone), and in our case, the disease responded with good clinical and imaging improvement of FDG avid lesions. Our findings suggest that combination chemoimmunotherapy may be a promising treatment option for composite lymphoma involving MCL and cHL, although further studies of this approach are warranted to provide definitive information for the treatment or to establish concrete guidelines for its management. The above report may give a hint on how best to treat composite lymphoma with concurrent MCL and cHL. Composite lymphomas should continue to be studied as each case of composite lymphoma represents a unique challenge and an opportunity to learn more about this complex disease.

## Conclusions

This case report describes a unique and extremely rare instance of composite lymphoma involving mantle cell lymphoma and classical HL. The rarity of this entity calls for further investigation into its pathogenesis and optimal treatment strategies. As seen in our case, combination chemoimmunotherapy holds promise for achieving remission in such cases, and continued research and clinical trials of composite lymphoma will contribute to a better understanding of this complex disease.
